# Development of the authentication and authorization processes for the iAgree portal, a platform for patient-controlled data sharing across health systems

**DOI:** 10.1093/jamiaopen/ooag111

**Published:** 2026-06-23

**Authors:** Spencer L Soohoo, Michelle Sophie Keller, Yunan Chen, Di Hu, Shao-Chi Huang, Tsung-Ting Kuo, Chloe Leder, Xi Lu, Daniela Meeker, Brad Morse, Harold Moyse, Gayathri Nagaraj, An T Nguyen, Lisa M Schilling, Andrey Soares, Mary A Whooley, Kai Zheng, Lucila Ohno-Machado

**Affiliations:** Enterprise Information Systems, Cedars-Sinai Medical Center, Los Angeles, CA, 90048, United States; Department of Computational Biomedicine, Cedars-Sinai Medical Center, Los Angeles, CA, 90048, United States; Leonard Davis School of Gerontology, University of Southern California, Los Angeles, CA, 90089, United States; Department of Medicine, Cedars-Sinai Medical Center, Los Angeles, CA, 90048, United States; Department of Informatics, Donald Bren School of Information and Computer Sciences, University of California, Irvine, CA, 92697, United States; Department of Informatics, Donald Bren School of Information and Computer Sciences, University of California, Irvine, CA, 92697, United States; Enterprise Information Systems, Cedars-Sinai Medical Center, Los Angeles, CA, 90048, United States; Department of Biomedical Informatics and Data Science, Yale School of Medicine, New Haven, CT, 06510, United States; Department of Surgery, Yale School of Medicine, New Haven, CT, 06520, United States; Leonard Davis School of Gerontology, University of Southern California, Los Angeles, CA, 90089, United States; Department of Information Science, University at Buffalo, New York, NY, 14260, United States; Department of Biomedical Informatics and Data Science, Yale School of Medicine, New Haven, CT, 06510, United States; Division of General Internal Medicine, Department of Medicine, University of Colorado Anschutz Medical Campus, Aurora, CO, 80045, United States; Enterprise Information Systems, Cedars-Sinai Medical Center, Los Angeles, CA, 90048, United States; Department of Biomedical Informatics and Data Science, Yale School of Medicine, New Haven, CT, 06510, United States; Department of Medicine, Cedars-Sinai Medical Center, Los Angeles, CA, 90048, United States; Department of Surgery, Yale School of Medicine, New Haven, CT, 06520, United States; Division of General Internal Medicine, Department of Medicine, University of Colorado Anschutz Medical Campus, Aurora, CO, 80045, United States; Department of Medicine, University of California, San Francisco, CA, 94143, United States; Department of Informatics, Donald Bren School of Information and Computer Sciences, University of California, Irvine, CA, 92697, United States; Department of Biomedical Informatics and Data Science, Yale School of Medicine, New Haven, CT, 06510, United States

**Keywords:** electronic health records, informed consent, privacy, federated identity, blockchain, patient portals, data sharing

## Abstract

**Objective:**

To develop the authentication and authorization module for iAgree, a privacy-preserving platform that enables patients to manage consent preferences and share electronic health record (EHR) data across multiple health systems with researchers. The consent and blockchain modules are described elsewhere.

**Materials and Methods:**

We developed the authentication and authorization module of iAgree. This module supports account creation and cross-institution identity binding as part of the iAgree workflow to enable patients to authenticate using federated credentials (eg, Google, Facebook). Patients link their identities across institutions by signing into each participating health system’s patient portal. Identity attributes retrieved via a Fast Healthcare Interoperability Resources (FHIR^®^) application programming interface (API) are cryptographically transformed into de-identified tokens so no raw identifiers are stored. Consent preferences are recorded immutably using blockchain technology. iAgree guides patients through account creation, cross-institution identity binding, and selection of granular data-sharing preferences. In this paper, we describe the design and implementation of the authentication and authorization modules, not the full workflow of the platform.

**Results:**

We installed the iAgree platform in a test or proof-of-concept environment at three health systems: Cedars-Sinai Medical Center, University of Colorado, and University of California, San Francisco. Functional testing with test patients demonstrated that authentication, identity binding, and secure multi-site record linkage could safely bind the identities of patients across sites, enabling patients to manage their data sharing consent preferences.

**Discussion:**

The results demonstrate the feasibility of a privacy-preserving multi-institutional data-sharing architecture that employs federated login, secure privacy-preserving multi-site record linkage, and blockchain-based consent tracking to align with privacy regulations while increasing transparency and patient autonomy.

**Conclusion:**

This effort demonstrated that the authentication and authorization module enables iAgree to be a feasible and secure platform for patient-directed sharing of EHR data across health systems with researchers. Future work will evaluate usability, patient engagement, and future real-world deployment.

## Background and significance

The secondary use of electronic health record (EHR) data has become a valuable resource for medical research and in most cases, unless patients explicitly opt-out, patient EHR data may be used for research without patients’ consent or knowledge.^1^^,^^2^ Even when patients provide informed consent to participate in a research study, they are often unaware of which specific data elements are shared, when the data is extracted, and whether or not it is shared with external institutions.^2^ Moreover, knowledge about opt-out procedures or policies is often low.^3^

Current approaches to obtaining informed consent via paper or electronically tend to be rigid and offer an all-or-nothing approach to data sharing. For example, a broad consent agreement might allow a patient’s entire medical record to be shared, while a more limited consent may restrict sharing to only a few types of data. Many health systems and countries also allow patients to opt-out of sharing de-identified EHR data for research.^4^ However, some patients might be more open to sharing their data if they had more control over which specific data types (eg, lab tests, demographics, diagnoses, genetic information) could be used and by whom (eg, researchers from nonprofit organizations, for-profit companies, or academic medical centers).^5–7^ The iCONCUR study explored the feasibility of offering patients more granular control over the sharing of their EHR data for research in a real-world setting.^8^ Patients participating in iCONCUR were presented with a tiered consent tool that allowed them to specify which of 37 data categories—such as demographics, socioeconomic status, sexuality, vital signs, disease conditions, treatments, laboratory results, social history, genetic information, and healthcare encounters—they were willing to share. They could also select the types of investigators who could access their data, choosing among researchers from nonprofit organizations, for-profit entities, and academic medical centers, including affiliated Veterans Affairs centers.

The results from iCONCUR indicated that patients appreciated having more detailed choices for data sharing, and they expressed a strong preference for information about who was using their data, the specific purposes of the research, and the outcomes of studies that utilized their information.^8^ On the other hand, a 2023 systematic review found that consent rates in general are lower when using opt-in procedures compared to opt-out procedures, and participants may be less representative of the study population when opt-in procedures are used.^9^

At the same time, regulatory and policy frameworks governing health data are evolving. In the United States, the Health Insurance Portability and Accountability Act (HIPAA) establishes privacy and security protections for health information through the HIPAA Privacy Rule and Security Rule. In Europe, the General Data Protection Regulation (GDPR) provides a broader framework governing the processing and sharing of personal data. While HIPAA primarily governs how covered entities manage and disclose health information, GDPR places stronger emphasis on individual control over personal data. Together, these policies emphasize the protection of personal health information and establish rights for individuals to access and control certain uses of their data. These evolving policy environments have important implications for the secondary use of electronic health record (EHR) data for research.^10^ However, existing data-sharing frameworks often struggle to support patient-mediated data sharing when patient information is distributed across multiple independent health systems or decentralized healthcare networks.

Moreover, high-profile data breaches and the misuse of personal information by third parties have contributed to a heightened concerns about data privacy,^11^ resulting in a demand for solutions that not only comply with regulatory requirements but also incorporate patient privacy in their architecture. Patients may be reluctant to consent to data sharing if they feel their data is not adequately protected. This may impede research progress and diminish the potential for discovering new treatments or improving healthcare delivery.^1^^,^^2^ A key challenge in health data sharing is to maintain patient trust while enabling researchers to access comprehensive datasets necessary for scientific advancement.^12^

Given these complexities, there is a need to empower patients to take an active role in managing how they share their health data for research. Systems for addressing these complexities must allow patients to customize their consent preferences, maintain control over how their information is shared across multiple health systems, and provide assurances that their data remains secure and private. Importantly, any solution must address the technical and ethical challenges of ensuring that once a patient sets their data-sharing preferences, these preferences are preserved and cannot be altered without their explicit consent.

The iAgree platform was developed to address the growing need for an approach that allows for data sharing while preserving privacy and allows patients to manage how that data is shared. We use a distributed architecture and blockchain technology designed to support secure, transparent, and tamper-resistant management of patient data-sharing preferences while minimizing exposure of direct identifiers. This design allows iAgree to meet regulatory standards and to address patients’ increasing concerns regarding privacy and control over their health data. We expect that iAgree would foster greater trust and encourage more active participation in health research.

While the iCONCUR study successfully demonstrated the feasibility of providing patients with granular control over data sharing within a single U.S.-based health system, it did not address the complexities that arise when patients receive care from multiple health systems.^8^ In many cases, patients have medical records spread across different institutions, especially if they have used primary care at one facility and sought specialty care at another. When participating in research, patients often only share data from one institution, which may not provide a comprehensive view of their medical history.

iAgree was designed to overcome this limitation by enabling the patients to manage data sharing preferences across multiple independent health systems. It was designed to give patients granular control over which specific types of EHR data are shared and with whom.

The objective of the larger project was to design and implement a privacy-preserving, patient-centered, web-based application, which enables individuals to manage consent preferences and securely share their EHR data sharing preferences across multiple independent health systems. This paper focuses on the design and implementation of the authentication and authorization (AA) modules for iAgree. In other related papers, we describe research and stakeholder preferences that led to the design of the iAgree platform,^12^ and a proof-of-concept study of a blockchain-based consent system aimed at protecting patient privacy while making it easier and more efficient for researchers to access approved data.^13^ Studies examining the usability of iAgree and simulations of studies aimed at examining how patients make decisions about sharing their medical data through the iAgree platform, focusing on the influence of study characteristics, privacy concerns, demographics, and perceived benefits on consent preferences are in progress.

Technical components of the web-based iAgree platform consist of:

1. An Authentication and Authorization (AA) module that allows patients to self-register an account to maintain their data sharing preferences across multiple health systems.
2. A consent module consisting of a web-based survey form to allow patients to express their data sharing preferences.
3. A blockchain module that uses smart contracts to ensure that consents and data sharing preferences are immutable.^13^

The focus of this paper is on the AA component of iAgree.

## Materials and methods

### Overall approach

The iAgree AA component was designed with a privacy-first approach, ensuring that patient confidentiality and security are at the forefront of its architecture. A key design consideration was to avoid storing any direct patient identifiers, such as names, Social Security Numbers, or other personally identifiable information. Instead, iAgree relies on a distributed system that uses the existing authentication protocols of health system patient portals that patients are familiar with. Once a patient has logged onto the portal, iAgree is allowed to securely access EHR data for transient use to construct encrypted tokens, thereby eliminating the need for centralized storage of sensitive identifiers. iAgree also includes a dashboard that provides patients with full visibility into the types of data being shared and identifies the researchers or organizations accessing their data.

### Blockchain technology

To maintain privacy while enabling data sharing, iAgree utilizes blockchain technology that provides a tamper-proof, immutable record of a patient’s data-sharing preferences, but without storing the actual health data or identifiable information. Instead, the blockchain records consent preferences, ensuring that these cannot be altered without patient approval, while still safeguarding the patient’s anonymity. More specifically, we adopted Ethereum^14^ and configured it as a permissioned blockchain,^15^ to which only allowed parties (iAgree sites in our design) have access, without needing a central intermediary. We used Proof-of-Authority (PoA)^16^ as our consensus mechanism to enable more efficient and scalable block creation processes under the more secured permissioned blockchain environment. We stored system-generated user IDs (one-way cryptographic hash of selected identifiers), study information, patients’ data sharing preferences, request information, and request decline reasons on-chain. The hashed IDs are used only internally in the iAgree system; the actual direct identifiers are not stored in any component of iAgree. We used Solidity^17^ to construct a smart contract with 19 functions to manage the on-chain data storage. When patients change their mind and would like to update (either agree or revoke) consents, we record all the transactions on-chain. Note that since we do not store patient data on-chain (and only store the consents), the revocation of the consents does not involve any data removal from the immutable blockchain. We demonstrated the feasibility of this approach in a prior publication.^13^

### Binding and authentication

The AA component of iAgree leverages the work we conducted as part of Single-FILE, a NIST-sponsored project aimed at exploring the feasibility of using a federated social credential to access multiple patient portals from one site.^18^ The AA component allows patients to select a social media identity (and corresponding login credentials) for management of consent preferences. For those who prefer not to use social media credentials, we also included an option to create a credential managed by the iAgree platform. Prior work shows that this can be safely done for healthcare.^18^ For this study, although we elected to use Facebook and Google as the identity providers, the architecture allows other identity providers to be used if they support OAuth2^19^ as part of their authentication protocol. This process is often referred to as delegated authentication, where one system relies on another to validate login credentials. This reduces the burden of remembering another set of credentials. Social credentials were selected since they are frequently used, although it is possible that with privacy concerns around the use of these social identity providers, certain individuals may prefer to sign up for iAgree using their preferred email address or one created specifically for iAgree. The system binds the social identity to the iAgree identity safely and accurately to the patient’s patient portal at each health system, thereby allowing the patient to self-manage the sharing preferences from a single portal. We implemented the iAgree platform at three health systems: Cedars-Sinai Medical Center (CS), University of Colorado (CU), and University of California, San Francisco (UCSF).

**Figure 1.**
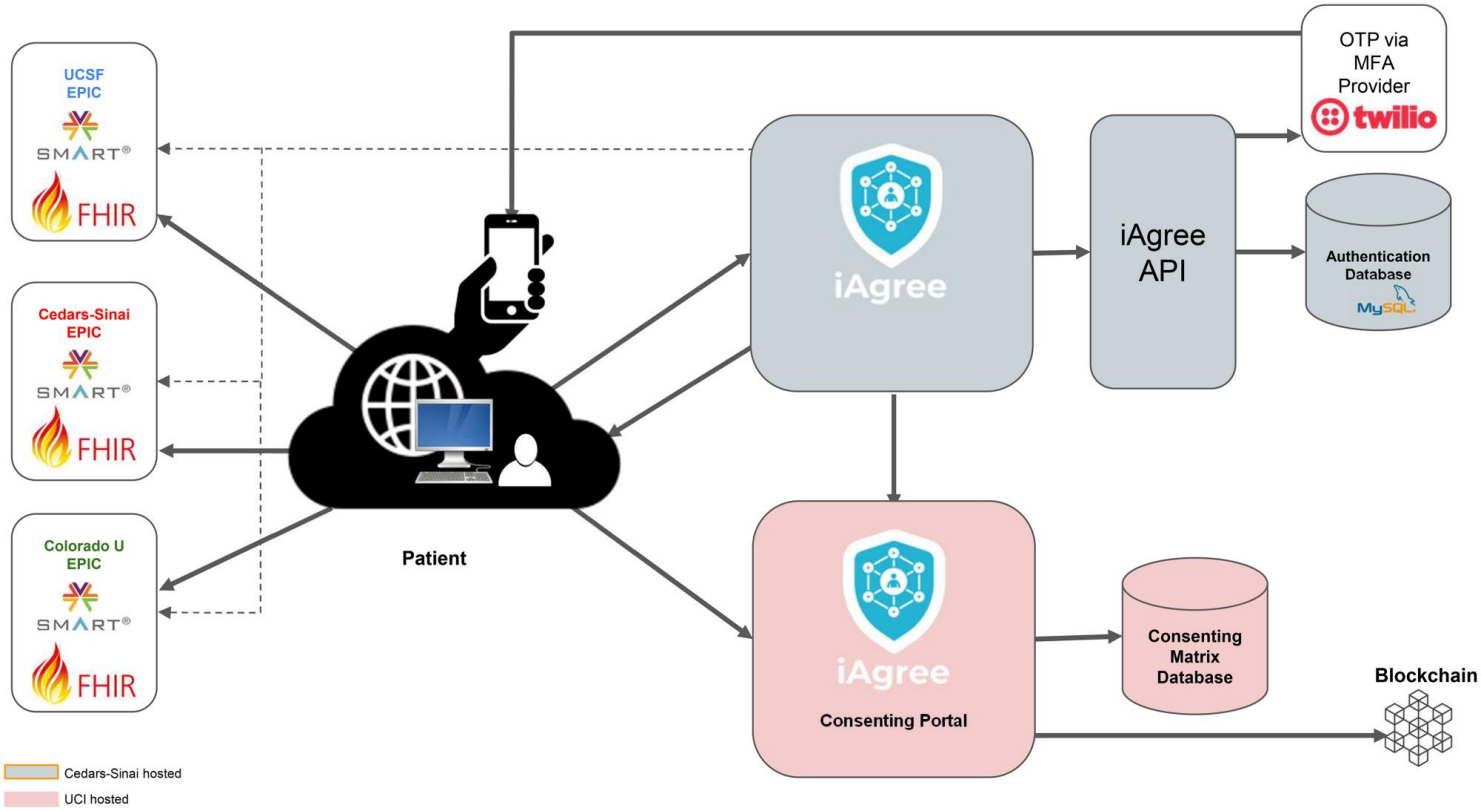
iAgree Architecture. This is a high-level view of the architecture for the iAgree platform. The iAgree platform authentication and authorization is hosted at CS and consents are hosted on a system at UCI where they are kept in a blockchain. When a patient registers in iAgree, SMART on FHIR is used to retrieve a phone number for identity verification via an OTP sent to the phone. This process is repeated for each health system where the patient wishes to share data for research, and iAgree uses one-way encrypted identifiers to link the patient at multiple health systems without storing any direct identifiers. After registration, the patient is routed to the iAgree consent portal to enter data sharing preferences, which are stored in a blockchain.

Figure 1 shows the overall architecture for the iAgree platform. A primary design constraint was to avoid storing any direct patient identifiers in the iAgree platform while providing a way to link patient identities in multiple, independent EHR systems. Our approach leverages the incorporation of patient portals and Substitutable Medical Applications and Reusable Technologies (SMART^®^) on Fast Healthcare Interoperability Resources (FHIR^®^) technology in EHR software.^20^ By having patient authentication occur using a health system’s EHR patient portal, iAgree does not need to manage any login credential and takes advantage of the security controls of the health system’s EHR system. Once a patient successfully authenticates into the EHR patient portal, a FHIR application programming interface (APIs) is used to exchange tokens that allow the iAgree platform to retrieve the patient identifiers listed in Table 1. These identifiers are used to (1) confirm that the login is legitimate (ie, not performed with stolen credentials) and (2) to create four unique identifiers based on a cryptographic hash of direct identifiers. The two actions performed are transactional, so only cryptographic hashes are stored by iAgree.

**Table 1.** Identifier combinations used for privacy-preserving record linkage.

Identifiers					
Firstname	Lastname	DOB	Sex	Mobilephone	Salt
Firstname	Lastname	DOB	Sex	Email	Salt
Soundex Firstname	Soundex Lastname	DOB	Sex	Mobilephone	Salt
Soundex Firstname	Soundex Lastname	DOB	Sex	Email	Salt

Each combination includes patient demographic information and a common (shared-secret) system-level salt value prior to hashing with Bcrypt. Soundex variations are included to account for minor spelling differences in names. Sex is normalized into a single character string, so either “M,” “F,” or “U.” Depending on the FHIR response/EMR, we will use the birthSex, legalSex, or genderIdentity in that order of priority.

Once patient identifiers are retrieved, a one-way cryptographic hash function, Bcrypt,^21^ is applied to the identifiers to create de-identified patient IDs. Bcrypt also adds a common (shared-secret), system-level salt^22^ to further mitigate the risk due to attacks. The resulting hashes enable the iAgree platform to recognize and link patient identities across multiple health systems while ensuring that no direct identifiers are stored or transmitted. This privacy-preserving mechanism is a key element of the iAgree architecture, enabling federated identity management without centralizing sensitive data.

**Figure 2.**
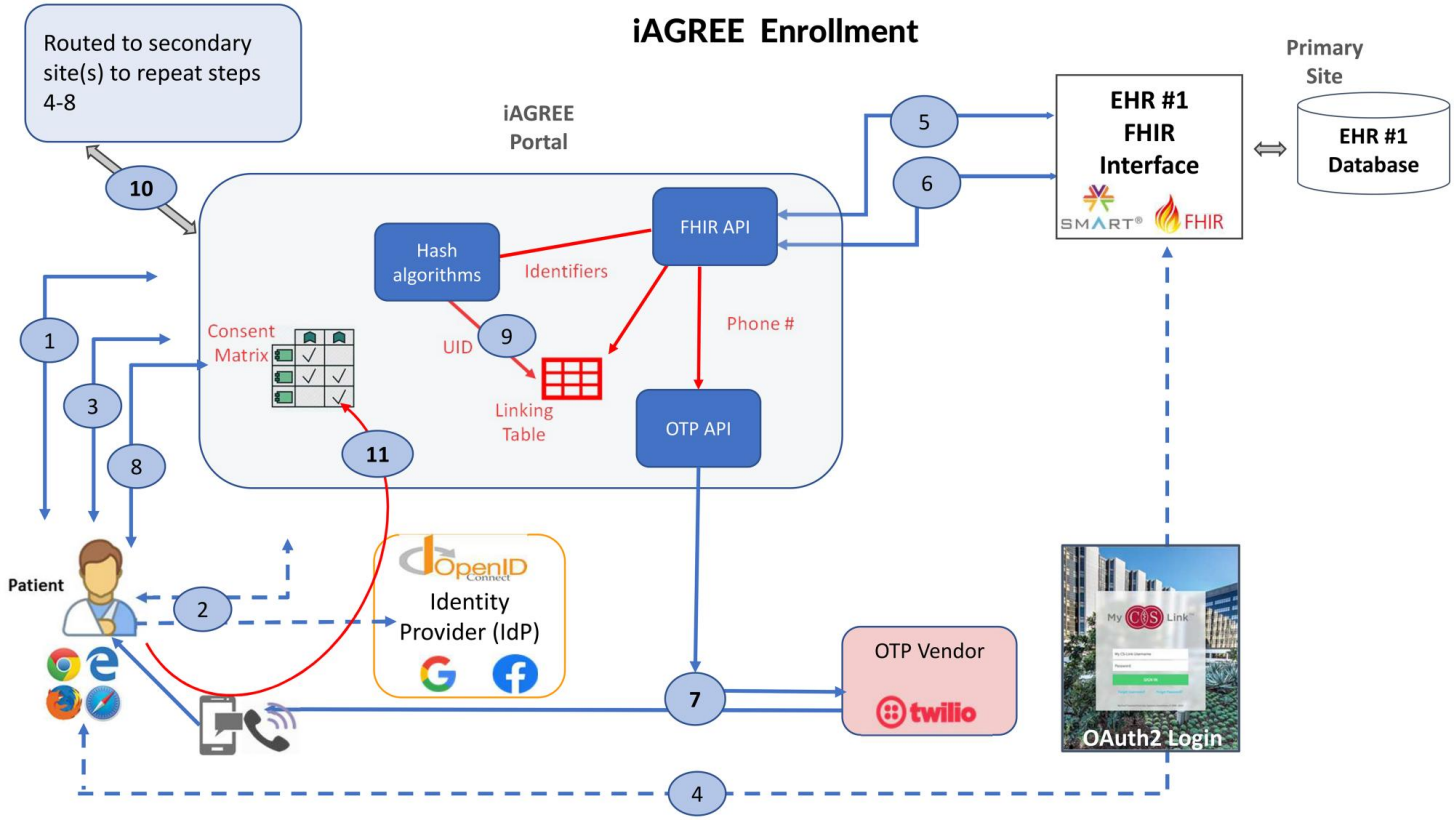
iAgree Patient Enrollment. Enrollment starts with accessing the iAgree portal and selecting an OpenID identity provider (Google or Facebook for this proof of concept) and logging in (Step 2). Step (3) is to select a primary health system, and the session is redirected to an OAuth2 log in for that site’s patient portal (Step 4). After a successful login, iAgree receives an OAuth2 patient token (Step 5), which is used to make a FHIR call on to retrieve the name, phone number, sex, date of birth, address, and FHIR ID (Step 6). The phone number is used to trigger an OTP (Step 7) that the patient confirms in Step 8. UIDs are constructed via a one-way salted hash of the identifiers, and along with the FHIR ID are stored in a Linking Table for future logins and to link with the data sharing preferences (Step 9). In Step 10, the patient is routed to a secondary health system to repeat Steps 4-9 for other sites. Step 11 captures the data sharing preferences via the consent module.

As illustrated in Figure 2, the iAgree identity binding process begins when a patient selects an identity provider—such as Facebook, Google, or iAgree itself—to log in to the platform. The patient then selects one or more participating health systems and authenticates to each system’s EHR patient portal, including completing a two-factor verification using a One-Time Passcode (OTP) sent to the phone number stored in the patient’s EHR demographics. This process is repeated for each health system. Once authenticated, iAgree uses Bcrypt to generate hashed identity keys from the retrieved patient identifiers. If a match is found between any of the hashed keys across different health systems, the patient’s iAgree identity is successfully linked. If no match is found, the patient is referred to a study coordinator for assistance in resolving any discrepancies.

Table 1 lists the combinations of patient identifiers that are hashed to generate cryptographic UIDs (User Identifiers) for secure record linkage. Each row represents a different combination of identifiers, including first name, last name, date of birth (DOB), sex, and either mobile phone number or email address. Two variations use standard text values, and two apply the Soundex phonetic algorithm to the name fields to allow for matching despite minor spelling variations. In all cases, a random string is used as a common (shared secret), system level salt that is added to the concatenated identifiers before encryption using Bcrypt. This helps prevent reverse-engineering and ensures uniqueness of the resulting hash. The *sex* field is normalized to a single character (“M,” “F,” or “U”) and is derived in priority order from the FHIR fields: birthSex, legalSex, or genderIdentity, depending on availability in the EHR system. For this proof of concept, simple deterministic matching of the encrypted UIDs was used. The modular design of the architecture allows possible replacement with a probabilistic matching algorithm for deployment in a real-world environment.

### Technical details

The iAgree authentication and authorization web component of iAgree was configured for deployment in a Docker container.^23^ The User Interface (UI) was built using ReactJS (a Javascript framework) with a NodeJS backend and interfaces with the iAgree API.

The iAgree API service was deployed as a Docker container in the Cedars-Sinai’s AWS Fargate environment. This API was built using the Jersey Framework (a Java-based framework) and interfaces with the database that is hosted in a Relational Data Base (AWS RDS) MySQL instance.

The Consenting module of the iAgree system is housed separately by the University of California, Irvine (UCI) and manages the consenting preferences of iAgree users who are authenticated via the authentication module.

JSON Web Tokens (JWT) tokens^24^ are exchanged in two separate ways to pass the authenticated user from the Authentication module to the Consenting module:

1. When a user logged into iAgree clicks a button to access the consent module, a JWT token is passed to the consent module as a URL parameter. The consent module server parses the URL parameter containing the JWT to extract the logged in user’s identity (email and system-generated UID). The JWT uses the HS256 algorithm and is signed with a 256-bit secret.
2. Every time there is a change made to an iAgree user account such as adding or removing a linked EHR identity (within the authentication module), a backend sync happens from the iAgree Authentication server to update the user changes into the iAgree Consenting server (the email and iAgree user ID are passed along with the list of FHIR IDs of the patient at each institution currently bound to the iAgree account).

### Alignment with privacy and regulatory standards

The design of the iAgree AA modules minimize risk to data by avoiding centralized storage of direct patient identifiers. Any retained information necessary for the system to function is encoded in salted, one-way hashes and system-generated identifiers (UIDs). Identifiers that are used to form the hashes are transient in nature and no longer accessible once a hash has been generated. This approach aligns with the core privacy and data minimization principles in regulations such as the HIPAA Privacy Rule and HIPAA Security Rule and the GDPR.

Patient autonomy is achieved via patient-initiated authentication and consent workflows; they can modify or revoke consent preferences over time, giving them total control over their consent preferences. Transparency is maintained via an immutable blockchain ledger that records all consent-related actions and enables verification of when and how consent was granted or changed. Consent revocability is implemented through an append-only consent model, ensuring that consent changes take effect immediately while preserving historical records.

Security safeguards include reliance on health system–managed authentication via EHR portals, OAuth2-based authorization, and signed JWTs to limit unauthorized access. This architecture addresses regulatory requirements while supporting scalable, multi-institutional research data sharing.

### Testing

We conducted structured functional testing of the iAgree AA module with twelve test patient accounts in Epic proof-of-concept environments at CS, CU, and UCSF. These accounts utilized two different social media identity providers (Facebook and Google), and testing was performed by volunteers who were not involved with the software development.

We used pre-defined cases to test matched salted-hashed identifiers at all three sites as well as single-identifier mismatches at one site. These cases included patients where all the identifiers in Table 1 were the same for patients at all three sites and cases where one of the identifiers (DOB, sex, or email) did not match at one of the sites.

## Results

We evaluated the iAgree AA component by creating test accounts with two social media identity providers (Facebook and Google) and test patient accounts in Epic test and proof-of-concept environments. Initially, this was done at CS and CU test environments. A third installation was performed at UCSF to expose and document potential configuration problems with a site that had not been involved with the early debugging of the software.

Functional testing confirmed that users could successfully complete the account creation, identity binding, and health system linking workflow illustrated in Figure 3. As shown in Figure 3a(1), users begin at the iAgree home page, where they can sign up using an existing Google or Facebook account or create a new account using their email. In Figure 3a(2), users have the option to enter their email address to receive notifications about research data sharing. The screenshot in Figure 3a(3) presents the interface for selecting participating health systems that maintain patient portals.

**Figure 3.**
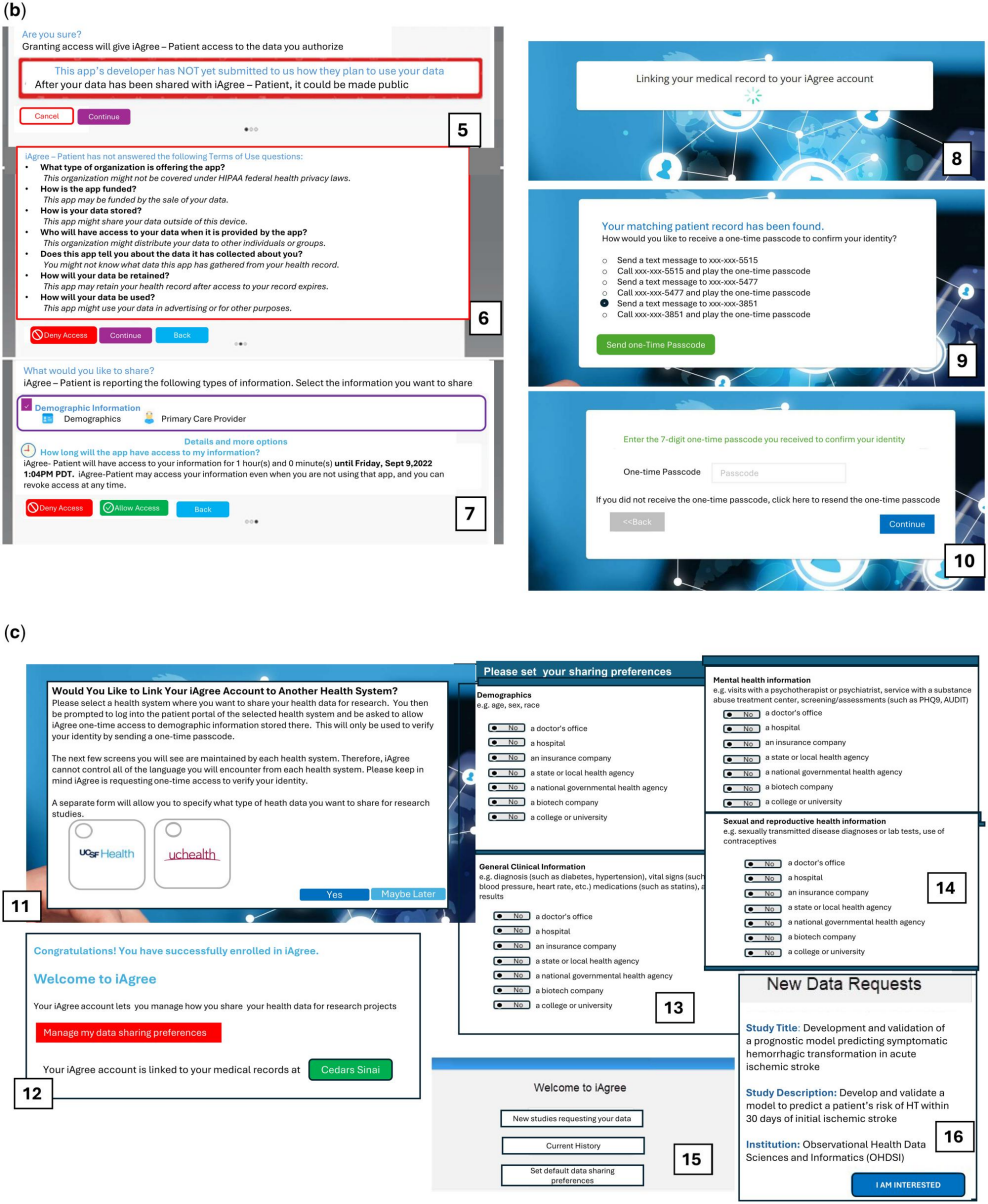
Continued.

**Figure 3.**
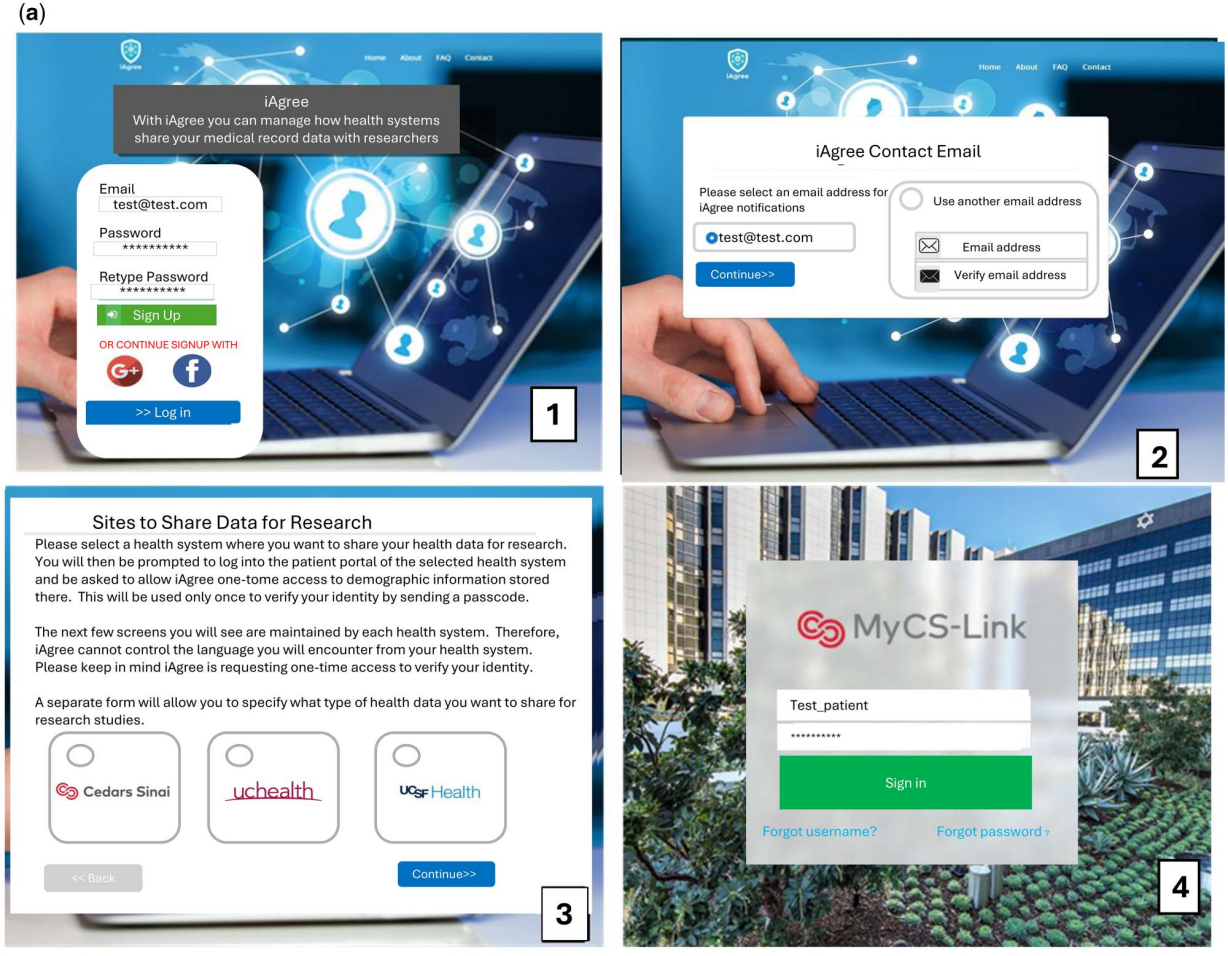
(a) Enrollment Screen Shots. This series of screen shots show the key user interface elements of the iAgree platform, which guide the user through account creation, identity verification, health system linking, and consent management. Screen [1] shows the iAgree home page, where users can sign up using a Google or Facebook account, or create an account with their own email. [2] Displays the option to enter an email address to receive notifications about research data sharing preferences. [3] Presents the interface where users select participating health systems with patient portals to link to their iAgree account for research consent management. [4] Shows the health system’s patient portal login screen, where users authenticate to verify their identity and securely bind their patient portal to their iAgree profile. (b) Identity Verification Screens. Screens [5] and [6] confirm access for the iAgree app to the patient data. [7] Highlights which data elements are shared with the iAgree app. [8] Confirms the binding. [9] Identifies the matching patient and directs the user to authenticate using 2-step authentication. [10] Continues the 2-step authentication process. (c) Final Enrollment Screens. Screen [11] allows the user to select another health system if desired where the process is repeated. [12] Shows the final enrollment screen for iAgree. [13] and [14] Show the options for selecting baseline data sharing preferences, and [15] shows the studies requesting data sharing and the user’s consent history. [16] Shows how data requests from different studies are presented to the user. Binding a patient identity to a health system means the patient is willing to share data from that site in response to a study participation request; no data is shared unless the patient explicitly consents for that study.

The next step is shown in Figure 3a(4) where users authenticate into a selected health system’s patient portal, initiating secure binding to their iAgree profile. Screens in Figure 3b(5)-(7) display the confirmation of data access permissions granted to the iAgree app, and details which specific data elements will be shared. The screens in Figure 3b(8)-(10) confirm the successful binding of the health system account and users are guided through a two-step authentication process (OTP) to further verify their identity. Users received an OTP on the phone number stored on the EHR. The final steps of the binding process are shown in Figure 3c(11) and (12) where users can repeat the process with additional health. After this point, the user session is redirected to the consent module hosted at UCI.

Figure 3c(13) and (14) are from the consent module and enable users to select baseline data sharing preferences. Screens in Figure 3c(15) and (16) display the history of studies to which they have consented and show how new data sharing requests from research studies are presented for user review and consent.

### Test results

Testing with identical (matched patients) and partially overlapping (un-matched patients) demographics showed that the system correctly prevented linkage and directed the patient to contact a study coordinator to resolve the demographic information discrepancy. For example, changing the date of birth so that CU was different from UCSF and CS resulted in a match for CS and UCSF, but not CU.

Testing also uncovered defects in the user interface, workflow, and redirection to the consent module. The defects were discussed at team meetings with the software developers and retesting performed to confirm that the defects had been corrected.

### Security assessment

The AA architecture ensured that no raw patient identifiers persisted within the iAgree platform. Only hashed system-generated UIDs were retained. However, we found it necessary to prompt patients to enter an email address as part of the account creation process to allow them to receive notifications of additional studies and system housekeeping messages. This could be an email address created specifically for iAgree notices, although in practice patients would likely use their preferred email address. Communication with the other iAgree modules relied on short-lived, signed JWTs; no protected health information, personally identifiable information, or consent data were stored within the AA module itself.

The addition of OTP verification for the EHR portal login provides assurance that the login is legitimate and is not performed with stolen credentials, so even if stolen credentials are being used for the social identity, the OTP serves as a barrier to binding the EHR identity.

The testing showed that the AA module functions were consistent with the privacy-preserving architecture. Specifically, we observed that when all salted-hashed identifiers matched at all sites the respective identities were bound, and binding was limited to matching sites when a single identifier did not match.

## Discussion

The iAgree platform addresses a critical gap in current health data-sharing infrastructures by providing patients with granular, privacy-preserving control over their EHR data across multiple health systems. This first phase of development demonstrates the feasibility of a system that integrates secure authentication, privacy-preserving record linkage, and user-centered interfaces to support federated identity management and consent tracking without storing patient identifiers.

Unlike existing approaches that often require patients to share all or none of their data, patients may use iAgree to tailor their data-sharing preferences by data type, research purpose, and institution. This capability builds upon insights from the iCONCUR study, which showed that patients prefer greater transparency and granular control when consenting to share their data for research.^8^ Importantly, iAgree extends these principles beyond a single health system to a multi-institutional environment, where patients’ data are often fragmented and access to comprehensive records is limited.

A key component of iAgree is the use of cryptographic one-way hashing to generate de-identified identity keys. By ensuring that no direct identifiers are stored on the platform, iAgree minimizes the risk of data breaches and unauthorized access, addressing a key concern in health data sharing while enabling accurate record linkage across multiple independent health systems without compromising patient privacy. This approach aligns with best practices in privacy-preserving technologies, directly addresses concerns about re-identification and data breaches,^25^^,^^26^ and builds trust with patients ensuring that their privacy is always respected. The architecture leverages existing FHIR-enabled patient portals and OAuth2-based federated authentication. This lessens the need for new credentials and reduces patient burden.

Figure 3 demonstrates that the user experience is intuitive and structured to guide patients through account creation, identity binding, preference setting, and consent management. Initial deployment demonstrated the feasibility of the approach used by iAgree for authentication and authorization. Future work will include user testing to evaluate usability, comprehension, and trust in the platform. As more patient portals move to requiring multi-factor authentication, the need to generate an OTP may be redundant, so future versions of iAgree may not require this step, thereby simplifying the account creation and binding process.

With evolving regulatory expectations and patient concerns around data privacy, platforms like iAgree have the potential to transform how health data is shared for research. While the portal was designed with patients through an extensive, iterative co-design process to minimize digital literacy barriers,^12^ future studies will assess the usability of iAgree among low digital literacy populations, alternative authentication methods besides social media credentials, and cultural concerns. Future research will assess the translation and cultural adaptation of iAgree for different populations, who may have specific privacy concerns. Longitudinal studies could examine whether providing patients with greater control over their data enhances trust in the research enterprise and improves recruitment and retention over time.

Research is also needed to explore the scalability of iAgree in larger, more complex health networks, including users from multiple EHR vendor systems, and to evaluate integration with emerging digital health tools, such as mobile health apps and patient-generated data platforms. We do not anticipate that integration with multiple EHR vendor systems will be problematic since the ONC 21st Century Cures Act Final Rule for EHR certification (45 CFR § 170.315(g)(10)) requires support for FHIR APIs and OAuth2 for authentication and authorization.

Prior to production use with actual patient data, the salted-hash algorithm will have to be evaluated to assess whether it meets the HIPAA expert determination standard for de-identification of patients. The design of the iAgree AA module allows for a replacement of the existing algorithm without any code changes. Also, information security penetration testing will have to be performed to surface vulnerabilities in the code.

One privacy compromise that had to be made was storing a contact email address in the iAgree platform. This was done so patients can receive notices such as system housekeeping alerts and notices about new research studies. One way to mitigate this privacy risk is to provide a notice at account creation time that patients can use a pseudonymous email address specifically for use with iAgree, albeit at the cost of another set of credentials to manage. Patients can perform their own risk/benefit assessment for this option.

Possible technological improvements include integration with blockchain-based researcher data request management platforms^26^ to further improve the completeness of the system. Finally, further technical work is warranted to refine the record linkage algorithms using probabilistic matching instead of deterministic matching^27^^,^^28^ and to explore interoperability with alternative consent models, including dynamic or just-in-time consent frameworks.

## Conclusion

The AA module of the iAgree platform successfully supported authentication and identity binding across three health systems and two external identity providers. It managed matched and un-matched test patient scenarios across all evaluated cases. This work demonstrates the feasibility of deploying a system that allows patients to securely manage their EHR data- sharing preferences across multiple, independent health systems. The platform integrates federated authentication, privacy-preserving record linkage, and blockchain-based consent tracking, thereby allowing patients to control which data types (if any) can be shared for research and to specify the types of research they are willing to participate in, while minimizing the risk of privacy breaches. The iAgree architecture aligns with core regulatory privacy and security elements and addresses patients’ increasing demand for transparency and autonomy. As iAgree advances to broader implementation and user testing, it holds promise as a scalable solution for ethical, privacy-conscious data sharing that supports high-quality, patient-authorized research.

## Data Availability

No patient-level data was collected for this study. Evaluation was limited to functional testing of the authentication and authorization components of iAgree. The resulting artifacts consist of test results and developer notes. These materials, including the source code, are not publicly available to avoid compromising the security of the system but may be available upon reasonable request, subject to institutional review and security assessment.
